# A Sustained Reduction in Serum Cholinesterase Enzyme Activity Predicts Patient Outcome following Sepsis

**DOI:** 10.1155/2018/1942193

**Published:** 2018-04-29

**Authors:** Aleksandar R. Zivkovic, Sebastian O. Decker, Anne C. Zirnstein, Annette Sigl, Karsten Schmidt, Markus A. Weigand, Stefan Hofer, Thorsten Brenner

**Affiliations:** ^1^Department of Anesthesiology, Heidelberg University Hospital, Heidelberg, Germany; ^2^Clinic for Anesthesiology, Intensive Care, Emergency Medicine I and Pain Therapy, Westpfalz Hospital, Kaiserslautern, Germany

## Abstract

Early sepsis identification is of paramount importance for an effective therapy and the patient outcome; however, a suitable prognostic biomarker is lacking. Anti-inflammatory nonneuronal cholinergic signaling modulates the magnitude of an immune response. Serum cholinesterase (BChE), an enzyme that hydrolyzes acetylcholine, plays an important role during inflammatory response and serves as an accurate index of cholinergic activity. BChE activity was measured in septic patients using a point-of-care system, and levels of conventional inflammatory markers and the disease severity scores were obtained. We observed a strong, sustained reduction in BChE activity in patients who died within a 90-day observation period, as compared to survivors. Reduced BChE activity when measured at the ICU admission effectively differentiated between the 90-day survivor and the nonsurvivor patient groups. We estimated a critical BChE level of 1.661 kU/L (CI 0.5–0.8, 94% sensitivity, 48% specificity, AUC 0.7) to best predict patient outcome providing a benchmark criterion for early detection of potentially fatal sepsis measured at the admission. This finding suggests that the BChE activity, used in combination with the laboratory tests, clinical examination, and the disease severity scoring, could serve to identify high-risk patients at the ICU admission, the most critical time point in the sepsis treatment.

## 1. Introduction

Sepsis, an infection complicated by acute organ dysfunction, is the major cause of death in the intensive care units, estimated to be far over 19 million cases per year worldwide [1]. Various factors, including the primary site of infection, the type of pathogen involved, the severity of the acute organ dysfunction, the primary health status, or the delay until the septic patient received the initial therapy, are decisive factors in the clinical appearance, severity, and prognosis of sepsis.

Despite improved modern critical care therapy including an early treatment initiation, support of failing organs, and treatment of the underlying infection, mortality lies between 20 and 30% in the last decade [2]. Early detection of the disease onset is therefore critical for the recognition of complex cases and high-risk patients who require immediate intensive medical care and appropriate therapy.

Various prognostic and diagnostic biomarkers for sepsis have been proposed in the literature [3]. Nevertheless, none of them show sufficient predictive power to be routinely employed in the clinical practice. Sepsis is an inflammatory response to multiple and extremely diverse factors. Therefore, identifying a single sepsis biomarker remains a challenge for critical care medicine.

Disease severity scoring systems (e.g., APACHE II, SOFA, and SAPS II) represent a quick and powerful complement to the laboratory biomarkers routinely used in the clinical setting [4, 5]. Clinical scores are a quick, noninvasive, and reliable assay used for patient stratification and outcome assessment in the ICU [6]. However, most of the scoring systems require a great amount of diverse patient data including laboratory and organ function tests, as well as a detailed patient history. In some cases, not all data is readily available at the most critical time point: the admission of a patient to the ICU with suspected septic onset.

The cholinergic system plays an important role in maintaining and modulating an adequate immune response upon an inflammatory episode. Cholinergic activity, acting extrasynaptically (nonneuronal acetylcholine), has been proposed to be a major mediator of the neuroimmune response to inflammation in a classical feedback reflex manner [7, 8]. Cholinergic activity is able to modulate the appropriate magnitude of an immune response upon an emerging inflammatory challenge [9–12]. The immune response might therefore be affected by the cholinergic system, acting anti-inflammatory.

Serum cholinesterase (butyrylcholinesterase (BChE)) is an enzyme that hydrolyzes acetylcholine [13]. BChE is synthetized in the liver and has conventionally been used as a liver function test. Our previous work, and that of others, has proposed that the enzymatic activity of the BChE might accurately reflect the nonneuronal cholinergic activity during systemic inflammation [14–20]. By using a point-of-care-testing (POCT) system, we have previously shown a rapid and strong reduction in the BChE activity following an inflammatory challenge [21]. Here, we tested whether the measured BChE activity, obtained from septic patients at the admission to the ICU, might predict the 90-day survival and therefore be used as an early assay for the septic patient outcome.

## 2. Materials and Methods

### 2.1. Study Design

This work represents a secondary analysis of the prospective observational study, powered for the detection of mycoses in patients with septic shock [22]. The study was performed at the intensive care unit of the Heidelberg University Hospital. The Ethics Committee of the Medical Faculty at the Heidelberg University approved this study (file number: S-097/2013). All patients or their legal designees gave informed consent. Patients included in this study were diagnosed septic according to the criteria described in the Surviving Sepsis Campaign: International Guidelines for Managements of Severe Sepsis and Septic Shock 2012 [23]. Exclusion criterion for this study was an extensively disrupted liver function with positive results in three or more of the following tests: aspartate aminotransferase (ASAT) > 100 U/L, alanine aminotransferase (ALAT) > 100 U/L, gamma glutamyl transferase (GGT) > 100 U/L, alkaline phosphatase (AP) > 200 U/L, total bilirubin > 2 mg/dL, and international normalized ratio (INR) > 1.3. Out of 50 recruited patients, 9 patients had documented extensive liver function disruption and were therefore excluded from the study. This resulted in a study population of 41 patients. Basic demographic data, site of infection, and the patient outcome are listed in Table 1.

### 2.2. Measurements

Blood samples used in this study were gathered at the time point of the sepsis detection, followed by blood samples collected 1, 2, 7, 14, 21, and 28 days later. Serum cholinesterase enzyme activity was measured by using the ChE check (Securetec Detektions-Systeme AG, Neubiberg, Germany; In-Vitro-Diagnostics Guideline 98/79/EG; DIN EN ISO 18113-2 and DIN EN ISO 18113-3) point-of-care-testing (POCT) device by following the manufacturer's instructions, as previously described in Zivkovic et al. [14]. Enzyme activity is measured in kU/L. The analysis of conventional inflammation biomarkers white blood cell count (WBCC), C-reactive protein (CRP), and procalcitonin (PCT) was conducted according to the standardized protocols of the central laboratory of the clinic. Plasma concentrations of interleukins IL-4, IL-6, and IL-10, tumor necrosis factor alpha (TNF-*α*), and midrange proadrenomedullin (MR-proADM) were measured according to the manufacturer's instructions as previously described [22]. Disease severity of septic patients was assessed at the ICU by obtaining APACHE II (Acute Physiology and Chronic Health Evaluation II), SOFA (Sequential Organ Failure Assessment), and SAPS II (Simplified Acute Physiology Score) scores. Patients were routinely scored at the above described time points for blood samples.

### 2.3. Statistical Analysis

The data were electronically gathered and stored by using Excel (Microsoft Corp., Redmond, WA). Data analysis was performed by using GraphPad Prism 6 for Mac (GraphPad Software, La Jolla California, USA, http://www.graphpad.com) and SPSS (version 21.0; SPSS Inc., Chicago, IL, USA). The optimal cutoff value estimation was performed by using Youden index. Data are presented as median with interquartile range (IQR). D'Agostino and Pearson omnibus normality test was used to verify the Gaussian distribution of the study groups. Statistical significance between the patient groups was tested using Mann–Whitney *U* test. A *p* value < 0.05 indicated statistical significance.

## 3. Results

**Figure 1:**
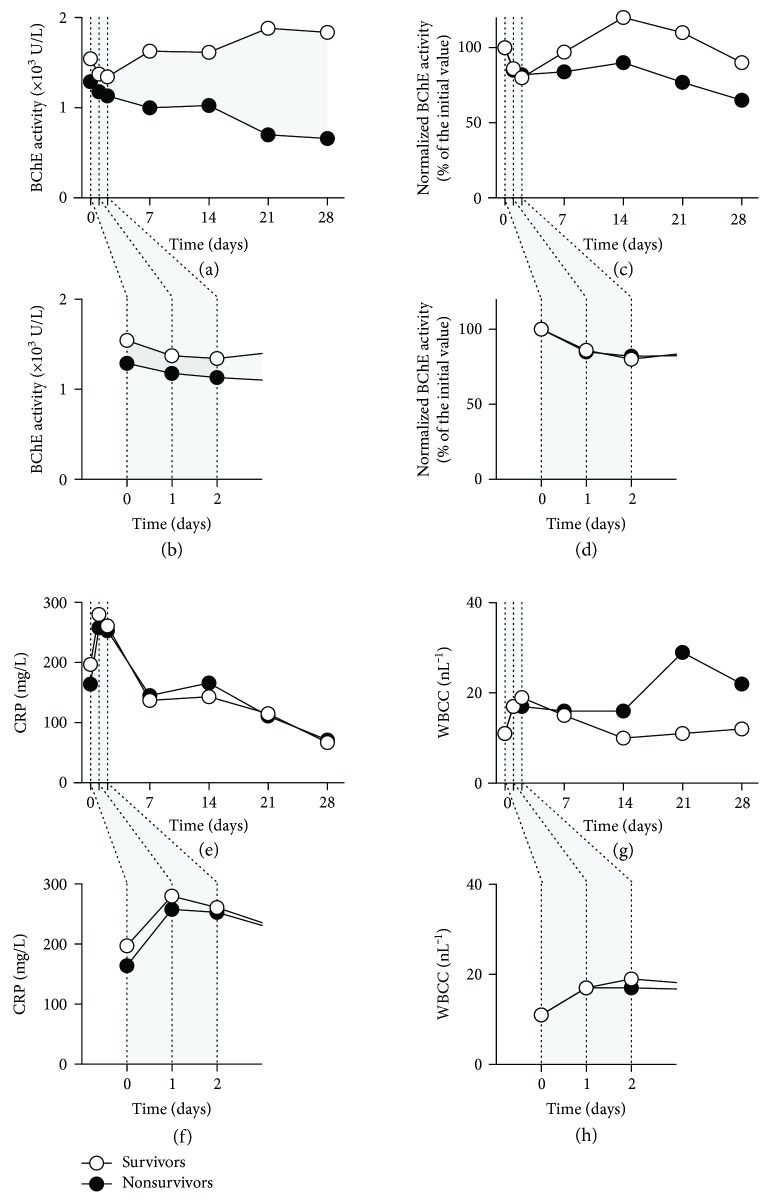
A sustained reduction in BChE enzyme activity identifies 90-day nonsurvivors following sepsis. BChE (a–d), CRP (e, f), and WBCC (g, h) measurements obtained from patient blood samples taken at the time point of the sepsis detection (0) and 1, 2, 7, 14, and 28 days later in patients surviving 90 days (open circles) and in nonsurvivors (closed circles). BChE enzyme activity is plotted as a raw value (a) and normalized to day 0 (initial value, c). Magnified time lines (b, d, f, h) show the measurements obtained during the first two days following sepsis detection. Dark gray-shaded areas indicate time periods where the statistical significance between the two patient groups occurs (*p* < 0.01, Mann–Whitney *U* test). Data are medians. BChE: butyrylcholinesterase; CRP: C-reactive protein; WBCC: white blood cell count.

We set the 90-day survival as a measure of the patient outcome following sepsis detection. Septic patients were divided in two groups: those who survived 90 days (survivors, 25 patients) and patients who did not survive 90 days following sepsis (nonsurvivors, 16 patients). Using the POCT, BChE enzyme activity was measured at the starting time point and on days 1, 2, 7, 14, 21, and 28 following the clinical onset of sepsis. As compared to the previously reported normal range [14], the BChE enzyme activity was markedly reduced in all septic patients during the observation period (*p* = 0.0001, Mann–Whitney *U* test), and the measured BChE activity in nonsurvivors was notably lower than that of the surviving patients for the duration of 28 days (*p* < 0.01, Mann–Whitney *U* test, Figures 1(a) and 1(b), Supplementary Figure 1, Supplementary Table 1). To be able to compare the BChE activity changes between patients, the enzyme activity was normalized to the initial value, obtained at the time point of the clinical onset of the sepsis. The analysis of the normalized BChE activity enabled the assessment of the amount and the rate of the enzyme activity changes during the observation period. The normalized BChE activity decreased within the first two days in both patient groups. During the following observation period, the normalized BChE activity of the survivors recovered, whereas the normalized BChE activity of the nonsurvivors remained strongly reduced (Figures 1(c) and 1(d), Supplementary Table 1). However, no statistical significance could be observed between the two patient groups. Concurrently, measured activity of the conventional inflammation biomarkers, CRP (Figures 1(e) and 1(f), Supplementary Table 1) and WBCC (Figures 1(g) and 1(h), Supplementary Table 1), showed an initial increase in activity, peaking two days after the clinical onset of the sepsis. No statistical difference between the surviving and the nonsurviving patients was observed.

**Figure 2:**
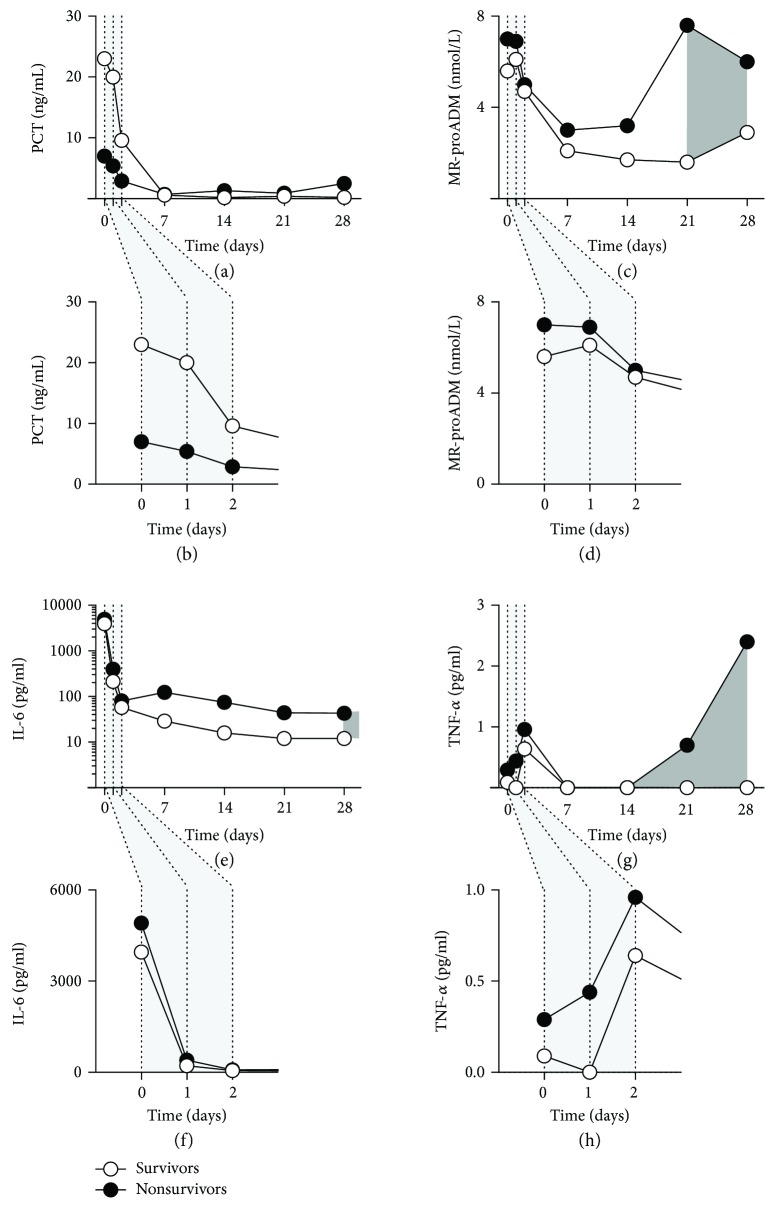
Activity pattern of the inflammatory biomarkers in patients with sepsis. Activity of PCT (a, b), MR-proADM (c, d), IL-6 (e, f), and TNF-*α* (g, h) was measured at the time point of sepsis detection (0) as well as 1, 2, 7, 14, 21, and 28 days later in patients who survived (open circles) and in those who died within 90 days (closed circles). Magnified time lines (b, d, f, h) represent measurements obtained within the first two days after the sepsis detection. Dark gray-shaded areas indicate time periods with the statistical difference between the patient groups (*p* < 0.05, Mann–Whitney *U* test). Data points are medians. PCT: procalcitonin; MR-proADM: midrange proadrenomedullin; IL-6: interleukin 6; TNF-*α*: tumor necrosis factor alpha.

Furthermore, we tested the activity pattern of PCT, a sepsis biomarker showing elevated activity during bacterial and fungal but not viral infections. Although increased PCT activity was initially observed in nonsurvivors, no differences were seen at the later time points (Figures 2(a) and 2(b), Supplementary Table 1). Significantly higher activity levels of MR-proADM, a novel sepsis biomarker, were observed in nonsurvivors in later time points starting 21 days following clinical onset of sepsis (*p* < 0.05, Mann–Whitney *U* test). However, MR-proADM activity levels could not discriminate between the surviving and the nonsurviving patients, when measured during the initial observation period (Figures 2(c) and 2(d), Supplementary Table 1). The activity pattern of the proinflammatory cytokines IL-6 and TNF-*α* as well as the anti-inflammatory cytokines IL-4 and IL-10 showed comparable activity patterns without a significant difference between the two patient groups in the initial observation period. The difference in the cytokine activities could be observed in the later time points after the clinical onset of the disease (after 28 days for IL-6, *p* < 0.05, Figures 2(e) and 2(f), Supplementary Table 1; after 14 days for TNF-*α*, *p* < 0.05, Figures 2(g) and 2(h), Supplementary Table 1; after 21 days for IL-4, *p* < 0.01, Supplementary Figure 2a, and 2b, Supplementary Table 2; and after 7 days for IL-10, *p* < 0.05, Supplementary Figure 2c, and 2d, Supplementary Table 2; Mann–Whitney *U* test). Thus, none of these conventional biomarkers could early predict patient survival at the 90-day period.

**Figure 3:**
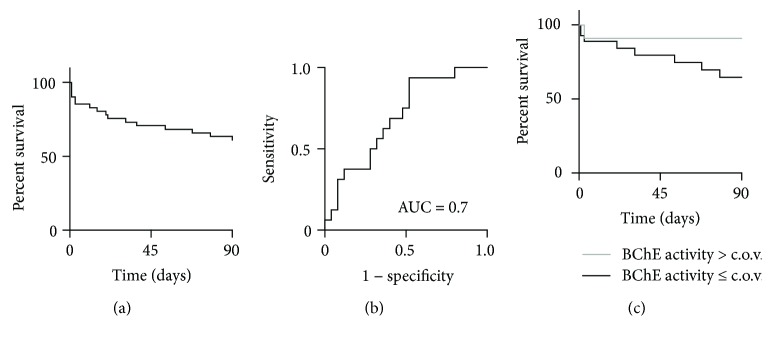
Measured BChE enzyme activity predicts the patient outcome following sepsis. (a) Kaplan–Meier curve shows the survival analysis of septic patients included in this study. (b) ROC curve analysis for the BChE activity measured at the time point of the clinical detection of sepsis revealed an AUC of 0.7 with the optimal cutoff value of 1.661 × 10^3^ U/L (CI 0.5–0.8, 94% sensitivity, 48% specificity). (c) A comparison of the Kaplan–Meier curves stratified according to the cutoff value for the measured BChE enzyme activity, as shown in (b). BChE: butyrylcholinesterase; ROC: receiver operating characteristic; AUC: area under the curve; CI: confidence interval; c.o.v.: cutoff value.

Next, we analyzed how well the measured BChE activity predicts the outcome of the septic patients. The 90-day survival of 61% following the clinical onset of the sepsis served as an independent long-term outcome parameter (Figure 3(a)). We observed that measured BChE enzyme activity could predict the survival of the septic patients. The receiver operator characteristic (ROC) curve analysis revealed an area under the curve (AUC) of 0.7. Further analysis rendered an optimal cutoff value of 1.661 kU/L for the measured BChE enzyme activity with 94% sensitivity and 48% specificity (Figure 3(b)). This finding was verified by plotting the Kaplan–Meier survival curves from septic patients with the initial BChE activity greater than the cutoff value and those with the initial BChE activity below the optimal cutoff value (Figure 3(c)). In addition, the ROC curve analysis of the conventional and novel sepsis biomarkers could poorly predict the outcome of the septic patients when measured at the initial time point (Supplementary Figure 3). We further examined the relationship between the patient outcome and the initially measured inflammation parameters (BChE, CRP, WBCC, PCT, MR-proADM, TNF-alpha, IL-4, IL-6, and IL-10) by conducting the *U* test and the full stepwise logistic regression analysis. Results of the *U* test (*p* = 0.04) and the stepwise logistic regression analysis (*p* = 0.03, likelihood ratio test) identified BChE as a sole independent prognostic factor for the patient outcome. Furthermore, we performed separate stepwise logistic regression analyses for three parameter subgroups. Separate analysis of the first subgroup (BChE, PCT, and MR-proADM) identified BChE enzyme activity as an independent prognostic factor for patient outcome (*p* = 0.02; likelihood ratio test). IL-10 was identified in the second subgroup (TNF-alpha, IL-10, and CRP) as an independent prognostic factor for patient outcome (*p* = 0.04; likelihood ratio test). In the third subgroup of factors (IL-4, IL-6, and WBCC), both IL-4 (*p* = 0.02, likelihood ratio test) and IL-6 (*p* = 0.045, likelihood ratio test) could be identified as independent prognostic factors for patient outcome.

BChE is synthetized in the liver, so impaired liver function would result in reduced BChE activity. To verify that reduced BChE activity is not caused due to liver dysfunction, we analyzed the liver function and found no impairment in septic patients included in the study (Supplementary Figure 4, Supplementary Table 3).

**Figure 4:**
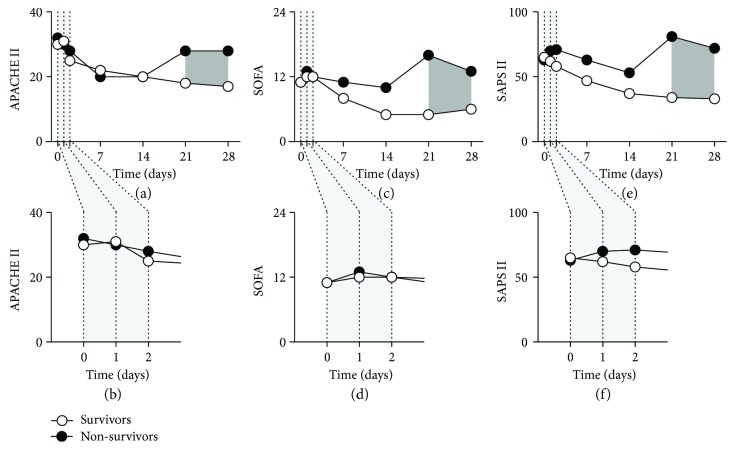
Disease severity scores predict outcome in patients with sepsis. APACHE II (a, b), SOFA (c, d), and SAPS II (e, f) scores were calculated from patients surviving sepsis (open circles) and from those who did not survive 90 days following sepsis detection (closed circles). Disease severity scores were obtained at the time point of the sepsis diagnosis and repeated 1, 2, 7, 14, 21, and 28 days later. Magnified time lines (b, d, f) show the disease severity scores obtained within the first two days following sepsis detection. Dark gray-shaded areas indicate the time points where the statistical significance could be observed (*p* < 0.05 for APACHE II, *p* < 0.01 for SOFA, and *p* < 0.001 for SAPS II; Mann–Whitney *U* test). Data points are medians. APACHE II: Acute Physiology and Chronic Health; SOFA: Sequential Organ Failure Assessment; SAPS II: Simplified Acute Physiology Score.

To assess the patient outcome following sepsis, we concurrently calculated the APACHE II and SOFA as well as SAPS II scores (Figure 4, Supplementary Table 1) for the survivor and the nonsurvivor patient groups. A difference between the two patient groups was observed 21 days following the clinical sepsis detection in all disease severity scores (APACHE II, *p* < 0.05, Figures 4(a) and 4(d), Supplementary Table 1; SOFA, *p* < 0.01, Figures 4(b) and 4(e), Supplementary Table 1; and SAPS II, *p* < 0.001; Mann–Whitney *U* test). However, the disease severity scores could not discriminate the nonsurvivors from the survivors when obtained at the initial time point.

## 4. Discussion

The present study highlights the validity and prognostic power of BChE measurements for early detection of high-risk, life-threatening sepsis. Reduced activity of the BChE, when measured at the clinical onset of sepsis, mirrors the activity changes of both conventional (CRP, WBCC, PCT) and novel (IL-4, IL-6, IL-10, TNF-*α*, MR-proADM) inflammation biomarkers and was validated as a biomarker in the early detection of sepsis by its correlation with several disease severity scores used for patient outcome analysis (APACHE II, SOFA, and SAPS II). Surprisingly, the BChE assessment at the onset of sepsis proved more effective than these disease severity scores in discriminating between the surviving and the nonsurviving patient groups making it a valuable biomarker for the early detection of high-risk sepsis patients.

In accordance with our previous studies, the reduction in BChE activity was observed as early as 1-2 hours following the inflammatory onset [21]. Moreover, a sustained BChE activity reduction, observed in nonsurviving patients, represents a novel red flag warning indicator in the critical care setting, allowing simple and rapid detection of the high-risk septic patients.

Septic patients included in the study showed elevated CRP and WBCC levels, which corresponded to the well-described pathophysiology following an immune response [24–30]. However, the initial measurements of these inflammatory biomarkers failed to distinguish between the survivor and the nonsurvivor patient groups in this study.

The levels of proinflammatory cytokines IL-6 and TNF-*α*, suggested to be effective in sepsis detection and mortality prediction in severe sepsis [31–36], showed altered, abnormal levels as expected throughout the observation period; however, these measurements during the initial 48 hours of observations could not discriminate the survivor and the nonsurvivor patient groups. We interpret this finding to be either due to the delayed abnormalities in cytokine peak activity (72 hours) [37], as compared to the earlier change in BChE activity, or due to the rather high variability of the obtained cytokine measurements. Moreover, separate subgroup stepwise logistic regression analysis identified IL-4, IL-6, and IL-10 as independent predictors for patient outcome, when measured at the initial time point. However, this finding could not be observed in other concurrently performed tests (*U* test, full stepwise regression analysis, Mann–Whitney *U* test). We interpret this observation to be presumably due to a low number of study patients and a high variability of the obtained interleukin measurements. Conducting a study with a larger population would presumably help overcome the observed discrepancy; however, such high variability suggests that cytokine tests will not provide a reliable biomarker assay for individual patient prognosis.

Our ROC analysis, identifying the optimal BChE cutoff value of the measured BChE enzyme activity at the time point corresponding to the clinical onset of sepsis, suggests that a single bedside BChE measurement could early identify a high-risk patient. Bedside measurement of BChE activity might help reduce the time delay between the initial clinical presentation of the patient with the suspected sepsis and the first therapeutic decisions, when conventional laboratory and diagnostic tests (e.g., chest X-ray and microbiology results) and clinical scores are not readily available. This is highly relevant for ICU clinicians who are often confronted with a fulminant course of the disease, requiring immediate action.

The AUC testing showed a reasonable accuracy for the BChE activity (0.7), which could be explained by the small group size in this study. The optimal cutoff value for the 90-day survival compares to the previously described BChE levels, observed upon the onset of the severe systemic inflammation [14]. Our previous study showed even stronger reduction of the normalized BChE activity upon systemic inflammation than nonnormalised data [21]; however, we did not observe this effect in the present study. Probable reason could be that the exact time point of the clinical diagnosis of the sepsis (and the time point of the initial BChE measurement) differed from the actual time point of the primary inflammation. The missed time interval between the genuine inflammation and the clinical onset of sepsis presumably led to a failed initial BChE measurement and the resulting initial activity reduction. This hypothesis could be further supported by the finding that the initially measured levels of the conventional inflammatory biomarkers (CRP, WBCC, PCT, IL-6, and TNF-*α*) showed markedly elevated activity, which are regularly described to peak at the earliest 24 hours following the inflammatory onset. This observation might therefore suggest that the proposed optimal cutoff value might be somewhat higher. Further study would be needed to answer this question.

MR-proADM, a peptide with immune modulating, metabolic, and vasoactive properties [38], has been shown to be an effective prognostic marker in sepsis [39] and septic shock [40] with a comparable and even superior prognostic value for sepsis relative to the conventional inflammatory biomarkers (CRP, PCT, and IL-6), as well as to the disease severity scores (APACHE II and SAPS) [39]. We observed a comparable pattern in BChE and MR-proADM activity when compared between the survivor and the nonsurvivor patient groups, further validating the efficacy of the BChE in predicting survival of patients with sepsis.

Disease severity scores are well-established clinical assays for the patient outcome analysis [6, 41–43]. In the present study, the difference between the two patient groups was revealed by the score calculations; however, these scores failed to discriminate the survivors from the nonsurvivors at the starting time point. These clinical scores are efficient tools in predicting outcome; however, they require documenting multiple and diverse datasets. The datasets are in most cases readily available; however, in some cases, a particular set of data might not be accessible, delaying or making the scoring impossible. By using a POCT system for a single BChE measurement, the results of an equally efficient outcome assessment tool are readily available at the bedside.

Why do nonsurviving septic patients show continuous and markedly lower levels of the serum cholinesterase enzymatic activity as compared to their surviving counterparts? Nonneuronal cholinergic activity is known to participate in the immune response [44]. The central nervous system (CNS) receives afferents from the immune system via both the humoral and the neural pathways [45, 46]. The humoral route uses the active transport of the proinflammatory cytokines through the blood-brain barrier, carrying the information about the commencing inflammatory process in the periphery. The neural immune pathway to the CNS from affected organs prevails when cytokine levels are low, providing early alert information of mild to moderate inflammation. This neural inflammatory signaling, together with pain and injury signals from the periphery, operates at a much lower detection threshold and reaches the CNS much quicker than the humoral pathway [12, 47]. The vagus nerve, a cholinergic neuron innervating most of the organs, mediates the neuroimmune response. The extrasynaptic cholinergic activity might therefore play a crucial role in both phases of the neuroimmune response: a quick and effective detection of an inflammatory challenge in the early phase but also in the process of maintaining the equilibrium of the neuroimmune response in the later phase of the severe inflammation. Therefore, the extrasynaptic activity of the cholinergic system might be of great importance during both early and late phases of the inflammatory response.

How does the enzyme activity of the serum cholinesterase relate to the cholinergic activity during an inflammatory episode? Several hypotheses might explain the observed phenomenon. Firstly, cholinergic neurotransmission is continuously active in a feedback response manner, maintaining physiological homeostasis. An inflammatory challenge would immediately cause cholinergic activation, causing a counteracting immune reaction [8]. The neuroimmune loop might act much quicker than the conventional immune reaction [48, 49]. The role of the reduced serum cholinesterase might mirror a neuroimmune setting where increased cholinergic activity is required. In the later time point, when the inflammation recedes and the organism recovers, cholinergic activity and the resulting levels of the serum cholinesterase will slowly increase, returning to the baseline. Such homeostatic regulation of BChE would presumably not take place in the continuously challenged and decompensated system of the nonsurvivor, where constantly low levels of the BChE activity mirror a decompensated organism which eventually leads to severe organ failure and death. The question whether low levels of BChE are caused by low levels of the extrasynaptically available enzyme substrate, acetylcholine, or a separate yet unknown mechanism reduces the BChE levels remains an open question. In the presence of low BChE activity, elevated levels of the extrasynaptic cholinergic activity would ensue, producing an anti-inflammatory response needed during the severe systemic inflammation. At this moment, it is not possible to provide a plausible answer whether reduced BChE activity results from the presumably reduced level of the nonneuronal acetylcholine or is actually an underlying cause. Further studies would be needed to address these questions.

Since serum cholinesterase is an enzyme synthetized in the liver [50, 51], the activity of the BChE is affected by disrupted liver function [52]. A clear limitation for using BChE enzyme activity as a biomarker for potentially fatal sepsis is patients with dysfunctional livers. The results obtained from these patients could not reliably be interpreted. Thus, the authors recommend against using this assay in patients with severe dysfunction of the liver.

A further limitation of this study is the low number of included patients. However, even with the low study sample, the described test demonstrated high sensitivity, particularly in the initial time period, as compared to the benchmark methods, suggesting a rapid, effective, and simple patient outcome assay. A larger, possibly multicenter, study would certainly be needed to validate our findings.

In summary, a single measurement of the BChE enzyme activity may serve as the simplest way to best predict patient outcome when performed at the initial time point. Moreover, the BChE activity correlates with the activity pattern of the conventional inflammatory biomarkers. The observed difference between the 90-day survivors and the nonsurvivors obtained by calculating the disease severity scores, a benchmark tool for the outcome analysis in the clinical setting, further verified the advantage of using the POCT method for a rapid patient outcome screening.

## 5. Conclusions

A single bedside measurement of the serum cholinesterase activity might be used to assess the 90-day patient outcome following sepsis. A suggested optimal cutoff value 1.661 kU/L, when obtained at the time point of the clinical onset of sepsis, might predict patient survival with 94% sensitivity and 48% specificity. This assay provides a rapid and simple bedside test which might be used for an early, simple, and effective identification of high-risk septic patients.

## Figures and Tables

**Table 1:** Study population demographics and clinical data.

*Patient data*	
Number of patients	41
Age (years)^∗^	66 (62–77)
Gender (male/female)	29/12
*Septic focus*	
Gastrointestinal tract	37 (90%)
Lung	8 (20%)
Genitourinary tract	1 (2%)
Other	2 (5%)
*Surgery*	
Patients subjected to the surgical revision	24 (59%)
*Length of stay*	
Days in the ICU^∗^	9 (4–18)
Days in the IMC^∗^	6 (0–14)
Days in the high dependence units (IMC + ICU)^∗^	18 (9–37)
Days in the hospital^∗^	40 (19–62)

ICU: intensive care unit; IMC: intermediate care unit. ^∗^Median with interquartile range.

## Supplementary Materials

Supplementary 1 Table 1: result summary.

Supplementary 2 Table 2: result summary for Supplementary Figure 2.

Supplementary 3 Table 3: result summary for Supplementary Figure 4.

Supplementary 4 Figure 1: the enzyme activity of the BChE in septic patients. Individual measurements (a, b) and median values (c, d) of the BChE enzyme activity obtained from 90-day survivors (open circles; a, c) and from patients who did not survive 90 days (closed circles, b, d) are obtained at the time point of the sepsis detection, 1, 2, 7, 14, 21, and 28 days later. Lines in (a) and (b) represent medians. Error bars in (c) and (d) are interquartile range.

Supplementary 5 Figure 2: activity pattern of the anti-inflammatory cytokines in septic patients. Activity of IL-4 (a, b) and IL-10 (c, d) was measured at the time point of sepsis detection (0) as well as 1, 2, 7, 14, 21, and 28 days later in patients who survived (open circles) and in those who died within 90 days (closed circles). Graphs (b) and (d) represent magnified time lines of the corresponding measurements obtained within the first two days after the sepsis detection. Data points are medians. IL-4: interleukin 4; IL-10: interleukin 10.

Supplementary 6 Figure 3: ROC curve analysis for the inflammatory biomarkers and disease severity scores. The ROC curves for the conventional inflammation biomarkers CRP, WBCC, and PCT are shown in panel (a). The ROC curves for MR-proADM, TNF-*α*, IL-4, IL-6, and IL-10 are shown in panel (b). The ROC analysis curves of the disease severity scores APACHE II, SOFA, and SAPS II are shown in panel (c). ROC: receiver operating characteristic; AUC: area under the curve; CRP: C-reactive protein; WBCC: white blood cell count; PCT: procalcitonin; MR-proADM: midrange proadrenomedullin; IL-4: interleukin 4; IL-6: interleukin 6; IL-10: interleukin 10; TNF-*α*: tumor necrosis factor alpha; APACHE II: Acute Physiology and Chronic Health; SOFA: Sequential Organ Failure Assessment; SAPS II: Simplified Acute Physiology Score.

Supplementary 7 Figure 4: liver function tests in septic patients. Median values of the ASAT, ALAT, GGT, and AP activities obtained from 90-day survivors (white) and from 90-day nonsurvivors (gray) are shown in the bar histogram (a). Histogram (b) shows median bilirubin and INR values obtained from the two patient groups. Shown data represent medians of the pooled values, collected at the previously described time points. Error bars are interquartile range. ASAT: aspartate aminotransferase; ALAT: alanine aminotransferase; AP: alkaline phosphatase; GGT: gamma glutamyl transferase; INR: international normalized ratio.

## References

[1] Adhikari N. K. J., Fowler R. A., Bhagwanjee S., Rubenfeld G. D. (2010). Critical care and the global burden of critical illness in adults.

[2] Angus D. C., Linde-Zwirble W. T., Lidicker J., Clermont G., Carcillo J., Pinsky M. R. (2001). Epidemiology of severe sepsis in the United States: analysis of incidence, outcome, and associated costs of care.

[3] Pierrakos C., Vincent J. L. (2010). Sepsis biomarkers: a review.

[4] Ghanem-Zoubi N. O., Vardi M., Laor A., Weber G., Bitterman H. (2011). Assessment of disease-severity scoring systems for patients with sepsis in general internal medicine departments.

[5] Sekulic A. D., Trpkovic S. V., Pavlovic A. P., Marinkovic O. M., Ilic A. N. (2015). Scoring systems in assessing survival of critically ill ICU patients.

[6] Vincent J. L., Moreno R. (2010). Clinical review: scoring systems in the critically ill.

[7] Andersson U., Tracey K. J. (2012). Neural reflexes in inflammation and immunity.

[8] Czura C. J., Tracey K. J. (2005). Autonomic neural regulation of immunity.

[9] Andersson U., Tracey K. J. (2012). Reflex principles of immunological homeostasis.

[10] Tracey K. J. (2009). Reflex control of immunity.

[11] Tracey K. J. (2007). Physiology and immunology of the cholinergic antiinflammatory pathway.

[12] Tracey K. J. (2002). The inflammatory reflex.

[13] Darvesh S., Hopkins D. A., Geula C. (2003). Neurobiology of butyrylcholinesterase.

[14] Zivkovic A. R., Schmidt K., Sigl A., Decker S. O., Brenner T., Hofer S. (2015). Reduced serum butyrylcholinesterase activity indicates severe systemic inflammation in critically ill patients.

[15] do Carmo G. M., Crivellenti L. Z., Bottari N. B. (2015). Butyrylcholinesterase as a marker of inflammation and liver injury in the acute and subclinical phases of canine ehrlichiosis.

[16] Zhang Q. H., Li A. M., He S. L. (2015). Serum total cholinesterase activity on admission is associated with disease severity and outcome in patients with traumatic brain injury.

[17] Distelmaier K., Winter M. P., Rützler K. (2014). Serum butyrylcholinesterase predicts survival after extracorporeal membrane oxygenation after cardiovascular surgery.

[18] Reid G. A., Chilukuri N., Darvesh S. (2013). Butyrylcholinesterase and the cholinergic system.

[19] Lampón N., Hermida-Cadahia E. F., Riveiro A., Tutor J. C. (2012). Association between butyrylcholinesterase activity and low-grade systemic inflammation.

[20] Ofek K., Krabbe K. S., Evron T. (2007). Cholinergic status modulations in human volunteers under acute inflammation.

[21] Zivkovic A. R., Bender J., Brenner T., Hofer S., Schmidt K. (2016). Reduced butyrylcholinesterase activity is an early indicator of trauma-induced acute systemic inflammatory response.

[22] Decker S. O., Sigl A., Grumaz C. (2017). Immune-response patterns and next generation sequencing diagnostics for the detection of mycoses in patients with septic shock—results of a combined clinical and experimental investigation.

[23] Dellinger R. P., Levy M. M., Rhodes A. (2013). Surviving sepsis campaign: international guidelines for management of severe sepsis and septic shock.

[24] Pepys M. B., Hirschfield G. M. (2003). C-reactive protein: a critical update.

[25] Welsch T., Frommhold K., Hinz U. (2008). Persisting elevation of C-reactive protein after pancreatic resections can indicate developing inflammatory complications.

[26] Castelli G. P., Pognani C., Cita M., Stuani A., Sgarbi L., Paladini R. (2006). Procalcitonin, C-reactive protein, white blood cells and SOFA score in ICU: diagnosis and monitoring of sepsis.

[27] Kobold A. C. M., Tulleken J. E., Zijlstra J. G. (2000). Leukocyte activation in sepsis; correlations with disease state and mortality.

[28] Tschaikowsky K., Hedwig-Geissing M., Braun G. G., Radespiel-Troeger M. (2011). Predictive value of procalcitonin, interleukin-6, and C-reactive protein for survival in postoperative patients with severe sepsis.

[29] Mortensen R. F. (2001). C-Reactive protein, inflammation, and innate immunity.

[30] Castelli G. P., Pognani C., Meisner M., Stuani A., Bellomi D., Sgarbi L. (2004). Procalcitonin and C-reactive protein during systemic inflammatory response syndrome, sepsis and organ dysfunction.

[31] Cohen J. (2002). The immunopathogenesis of sepsis.

[32] Blackwell T. S., Christman J. W. (1996). Sepsis and cytokines: current status.

[33] Dama P., Ledoux D., Nys M. (1992). Cytokine serum level during severe sepsis in human IL-6 as a marker of severity.

[34] Gogos C. A., Drosou E., Bassaris H. P., Skoutelis A. (2000). Pro- versus anti-inflammatory cytokine profile in patients with severe sepsis: a marker for prognosis and future therapeutic options.

[35] Oberholzer A., Souza S. M., Tschoeke S. K. (2005). Plasma cytokine measurements augment prognostic scores as indicators of outcome in patients with severe sepsis.

[36] Bozza F. A., Salluh J. I., Japiassu A. M. (2007). Cytokine profiles as markers of disease severity in sepsis: a multiplex analysis.

[37] Andaluz-Ojeda D., Bobillo F., Iglesias V. (2012). A combined score of pro- and anti-inflammatory interleukins improves mortality prediction in severe sepsis.

[38] Linscheid P., Seboek D., Zulewski H., Keller U., Müller B. (2005). Autocrine/paracrine role of inflammation-mediated calcitonin gene-related peptide and adrenomedullin expression in human adipose tissue.

[39] Christ-Crain M., Morgenthaler N. G., Struck J., Harbarth S., Bergmann A., Müller B. (2005). Mid-regional pro-adrenomedullin as a prognostic marker in sepsis: an observational study.

[40] de la Torre-Prados M. V., Garcia-De La Torre A., Enguix A., Mayor-Reyes M., Nieto-González M., Garcia-Alcantara A. (2016). Mid-regional pro-adrenomedullin as prognostic biomarker in septic shock.

[41] Knaus W. A., Draper E. A., Wagner D. P., Zimmerman J. E. (1985). APACHE II: a severity of disease classification system.

[42] Le Gall J. R., Lemeshow S., Saulnier F. (1993). A new Simplified Acute Physiology Score (SAPS II) based on a European/North American multicenter study.

[43] Vincent J. L., Moreno R., Takala J. (1996). The SOFA (Sepsis-related Organ Failure Assessment) score to describe organ dysfunction/failure.

[44] Wang H., Yu M., Ochani M. (2003). Nicotinic acetylcholine receptor alpha7 subunit is an essential regulator of inflammation.

[45] Blalock J. E. (2005). The immune system as the sixth sense.

[46] Blalock J. E. (1984). The immune system as a sensory organ.

[47] Borovikova L. V., Ivanova S., Zhang M. (2000). Vagus nerve stimulation attenuates the systemic inflammatory response to endotoxin.

[48] Goehler L. E., Gaykema R. P. A., Hansen M. K., Anderson K., Maier S. F., Watkins L. R. (2000). Vagal immune-to-brain communication: a visceral chemosensory pathway.

[49] Pavlov V. A., Wang H., Czura C. J., Friedman S. G., Tracey K. J. (2003). The cholinergic anti-inflammatory pathway: a missing link in neuroimmunomodulation.

[50] Pohanka M. (2013). Butyrylcholinesterase as a biochemical marker.

[51] Santarpia L., Grandone I., Contaldo F., Pasanisi F. (2013). Butyrylcholinesterase as a prognostic marker: a review of the literature.

[52] Meng F., Yin X., Ma X., Guo X.-D., Jin B., Li H. (2013). Assessment of the value of serum cholinesterase as a liver function test for cirrhotic patients.

