# Supplementary material for: A Sustained Reduction in Serum Cholinesterase Enzyme Activity Predicts Patient Outcome following Sepsis
Source: Mediators Inflamm. 2018 Apr 29;2018:1942193. doi: 10.1155/2018/1942193 (PMC5949165; doi:10.1155/2018/1942193)
Supplement: Supplementary 1 — Table 1: result summary. [file 1942193.f1.docx]

| **Supplementary Table 1** | | | | | | | | | | | | |
| --- | --- | --- | --- | --- | --- | --- | --- | --- | --- | --- | --- | --- |
| **Figure 1** | | | | | | | | | | | | |
| median (IQR) | | starting time point (sepsis detection) | day 1 | day 2 | | day 7 | | day 14 | | day 21 | | day 28 |
| BChE activity  (x10^3^ U/L) | 90-day survivors | 1.544 (1.173-2.007) | 1.373 (1.115-1.719) | 1.342 (1.104-1.709) | | 1.627 (1.2-1.987) | | 1.614 (1.153-2.187) | | 1.882 (1.415-2.08) | | 1.837 (1.242-2.582) |
|  | 90-day non-survivors | 1.289 (0.935-1.553) | 1.177 (0.804-1.414) | 1.131 (0.939-1.267) | | 0.999 (0.731-1.22) | | 1.026 (0.637-1.499) | | 0.699 (0.442-1.091) | | 0.659 (0.27-0.943) |
| normalized BChE activity  (% of initial value) | 90-day survivors | / | 86 (74-100) | 80 (74-100) | | 97 (72-120) | | 120 (71-130) | | 110 (78-120) | | 90 (89-150) |
|  | 90-day non-survivors | / | 85 (73-99) | 82 (74-92) | | 84 (62-110) | | 90 (56-120) | | 77 (29-110) | | 65 (32-94) |
| CRP (mg/L) | 90-day survivors | 197 (154-291) | 280 (219-338) | 261 (198-362) | | 137 (101-181) | | 143 (76-157) | | 115 (104-147) | | 67 (40-172) |
|  | 90-day non-survivors | 164 (72-338) | 258 (197-306) | 253 (150-297) | | 145 (107-203) | | 166 (59-198) | | 111 (63-152) | | 71 (64-130) |
| WBCC (nl^-1^) | 90-day survivors | 11 (3-18) | 17 (10-24) | 19 (11-27) | | 15 (12-20) | | 10 (9-13) | | 11 (9-14) | | 12 (10-17) |
|  | 90-day non-survivors | 11 (6-21) | 17 (11-32) | 17 (10-26) | | 16 (10-28) | | 16 (9-21) | | 29 (17-34) | | 22 (12-27) |
|  | | | | | | | | | | | | |
| **Figure 2** | | | | | | | | | | | | |
| PCT (ng/mL) | 90-day survivors | 23 (7-43) | 20 (5-43) | | 10 (3-23) | | 0.59 (0.31-2) | 0.16 (0.09-0.64) | 0.41 (0.1-0.89) | | 0.22 (0.1-0.4) | |
|  | 90-day non-survivors | 7 (4.5-26) | 5.4 (2.8-71) | | 2.9 (1.7-10) | | 0.69 (0.46-4.1) | 1.3 (0.18-4.2) | 0.89 (0.05-8) | | 2.5 (1.3-3.7) | |
| MR-proADM (nmol/L) | 90-day survivors | 5.6 (4.4-9.1) | 6.1 (3.8-9.8) | | 4.7 (2.8-7.7) | | 2.1 (1.4-3.6) | 1.7 (1.0-2.9) | 1.6 (1.2-3.9) | | 2.9 (1.0-5.5) | |
|  | 90-day non-survivors | 7 (5-11) | 6.9 (3.7-10) | | 5.0 (3.1-6.3) | | 3.0 (1.9-4.9) | 3.2 (1.3-6.0) | 7.6 (4.5-11.0) | | 6.0 (4.5-13.0) | |
| IL-6 (pg/ml) | 90-day survivors | 3961 (1293-15969) | 214 (102-499) | | 58 (25-162) | | 29 (12-119) | 16 (4.6-127) | 12 (7-20) | | 12 (6-21) | |
|  | 90-day non-survivors | 4913 (1256-184922) | 398 (119-2963) | | 80 (55-1382) | | 123 (50-255) | 75 (12-269) | 44 (13-1069) | | 43 (31-413) | |
| TNF-α (pg/ml) | 90-day survivors | 0.09 (0-1.1) | 0 (0-1.6) | | 0.64 (0-1.9) | | 0 (0-0) | 0 (0-0) | 0 (0-0) | | 0 (0-0) | |
|  | 90-day non-survivors | 0.29 (0-2.1) | 0.44 (0-2.4) | | 0.96 (0-3.6) | | 0 (0-0.2) | 0 (0-0.26) | 0.7 (0.15-2.4) | | 2.4 (0.17-4.4) | |
|  | | | | | | | | | | | | |
| **Figure 4** | | | | | | | | | | | | |
| APACHE II score | 90-day survivors | 30 (26-34) | 31 (27-33) | | 25 (22-32) | | 22 (17-27) | 20 (13-22) | 18 (15-23) | | 17 (15-25) | |
|  | 90-day non-survivors | 32 (29-38) | 30 (27-38) | | 28 (24-38) | | 20 (13-35) | 20 (11-28) | 28 (23-36) | | 28 (19-33) | |
| SOFA score | 90-day survivors | 11 (10-14) | 12 (11-14) | | 12 (10-14) | | 8 (4-11) | 5 (2-8) | 5 (1-8) | | 6 (4-9) | |
|  | 90-day non-survivors | 11 (9-14) | 13 (11-14) | | 12 (10-15) | | 11 (4-14) | 10 (1-14) | 16 (10-20) | | 13 (11-19) | |
| SAPS II score | 90-day survivors | 65 (44-72) | 62 (48-78) | | 58 (42-70) | | 47 (30-58) | 37 (23-49) | 34 (16-43) | | 33 (24-55) | |
|  | 90-day non-survivors | 63 (49-78) | 70 (57-84) | | 71 (50-79) | | 63 (33-71) | 53 (31-59) | 81 (55-102) | | 72 (46-90) | |
