# Supplementary material for: A Sustained Reduction in Serum Cholinesterase Enzyme Activity Predicts Patient Outcome following Sepsis
Source: Mediators Inflamm. 2018 Apr 29;2018:1942193. doi: 10.1155/2018/1942193 (PMC5949165; doi:10.1155/2018/1942193)
Supplement: Supplementary 2 — Table 2: result summary for Supplementary Figure 2. [file 1942193.f2.docx]

| **Supplementary Table 2** | | | | | | | | |
| --- | --- | --- | --- | --- | --- | --- | --- | --- |
| median (IQR) | | starting time point (sepsis detection) | day 1 | day 2 | day 7 | day 14 | day 21 | day 28 |
| IL-4  (pg/mL) | 90-day survivors | 0 (0-0.7) | 0 (0-1.1) | 0 (0-1.2) | 0 (0-0.02) | 0 (0-0.03) | 0 (0-0) | 0 (0-0.02) |
|  | 90-day non-survivors | 0.12 (0-1.6) | 0 (0-1.8) | 0.06 (0-1.9) | 0 (0-0.38) | 0 (0-0.45) | 0.3 (0.073-1.1) | 1.1 (0.49-1.8) |
| IL-10  (pg/mL) | 90-day survivors | 19 (5.7-208) | 5.3 (2.8-8.6) | 3.5 (1.2-6) | 0.46 (0.19-1.2) | 0.25 (0.095-0.62) | 0.18 (0-0.41) | 0.28 (0.04-0.42) |
|  | 90-day non-survivors | 15 (5.7-156) | 6 (1.8-19) | 4.2 (1.6-7.5) | 1.3 (1-2.5) | 1.2 (0.94-1.3) | 1.9 (1.1-3.8) | 3 (1.3-3.4) |
