# Supplementary material for: A Sustained Reduction in Serum Cholinesterase Enzyme Activity Predicts Patient Outcome following Sepsis
Source: Mediators Inflamm. 2018 Apr 29;2018:1942193. doi: 10.1155/2018/1942193 (PMC5949165; doi:10.1155/2018/1942193)
Supplement: Supplementary 3 — Table 3: result summary for Supplementary Figure 4. [file 1942193.f3.docx]

| **Supplementary table 3** | | | | | | |
| --- | --- | --- | --- | --- | --- | --- |
| median (IQR) | ASAT (U/L) | ALAT (U/L) | AP (U/L) | GGT (U/L) | bilirubin (mg/dL) | INR |
| 90-day  survivors | 39 (23-65) | 23 (16-41) | 99 (65-150) | 60 (27-144) | 0.4 (0.2-1.0) | 1.1 (1.0-1.2) |
| 90-day  non-survivors | 60 (33-172) | 44 (31-96) | 99 (71-164) | 76 (42-151) | 0.8 (0.5-3.2) | 1.2 (1.1-1.4) |
